# Attitudes Towards Appearance and Body-Related Stigma Among Young Women With Obesity and Psoriasis

**DOI:** 10.3389/fpsyt.2021.788439

**Published:** 2021-11-11

**Authors:** Natalia Mazurkiewicz, Jarosław Krefta, Małgorzata Lipowska

**Affiliations:** ^1^Institute of Psychology, University of Gdańsk, Gdansk, Poland; ^2^Creative Code Studio, Gdynia, Poland

**Keywords:** body attitude, body image, body stigma, obesity, skin disease

## Abstract

The goal of this study was to investigate the role of the subjective assessment of one's body image in the relationship between objective indices of appearance and perceived stigma in young women affected by obesity and psoriasis. These are chronic diseases that decrease one's physical attractiveness and are associated with stigmas related to body defects. A total of 188 women in early adulthood took part in the study (*M* = 25.58; *SD* = 2.90), including obese women (*n* = 54), women suffering from psoriasis (*n* = 57), and a control group (*n* = 77). The participants completed the Multidimensional Body-Self Relations Questionnaire, Perceived Stigmatisation Questionnaire, and a socio-demographic questionnaire. Anthropometric data were gathered using a body composition analyzer. Objective parameters of body shape were calculated (WHR and ICO). Subjective assessment of one's body and attitudes towards one's body were found to influence perceived stigma, independently of the condition causing the stigma and of the objective appearance of the participant. This study did not support the existence of a relationship between parameters regarding body shape and sense of stigma, even when subjective body assessment acted as a moderator. At the same time, body mass was a strong predictor of levels of perceived stigma. Women affected with obesity perceived a higher level of stigma than the other groups. The severity of psoriasis did not impact the perceived stigma. Moreover, women with psoriasis assessed their health—as a part of the assessment of their bodies—the highest, which may explain the lower perceived stigma in this group.

## Introduction

Body image, assessment of one's body, and attitudes towards one's body are important elements of the development of the “self” (1). Research suggests that how we perceive our body is more important for our body image than its actual appearance (2, 3). This is important, because one's body image and sense of attractiveness are strongly associated with self-esteem (4, 5), well-being (6), happiness (7, 8), life satisfaction (9), and it influences health-related quality of life (10–12). Our attitudes towards our bodies are shaped and change throughout the different stages of our lives. Assessment of one's body decreases significantly during adolescence and early adulthood—mainly because of judgement from one's environment (13–15)—increases slowly during mid-adulthood (16), and then rapidly drops when one reaches old age (17, 18). People tend to be most critical of their bodies during adolescence and young adulthood (19), and the imperfections typical of teenage bodies are often used by peers as a source of mockery or rejection (20, 21). The shaping of one's body image is influenced by attitudes and beliefs regarding one's body (subjective opinions and perceptions, such as satisfaction or dissatisfaction with one's looks), physical factors (objective body measurements, proportions, body mass, height), interpersonal factors (opinions from one's environment; e.g., family members, peers), and cultural factors (e.g., from the media) (1, 22). The latter have a huge influence over the “ideal” an individual tries to attain, because they play a significant role in the acceptance and construction of standards for bodies (23). Western culture promotes body ideals characterised by tall, slim female bodies (24, 25), with constant breasts-to-waist ratio and a low hip-to-waist ratio (26, 27), while perfect male bodies are muscular and mesomorphic (V-shaped, with broad shoulders and a narrow waist) (28, 29).

Thus, the way a person views their body depends not only on their culture (30–33) and age (16, 17), but also on their gender (34). Body image is a much more important part of the “self” for girls and women than it is for men, and thus it directly and strongly shapes their overall levels of self-esteem (35, 36). In comparison to men, women are more prone to negative body-image evaluations and pay more attention to their looks (37); furthermore, they are subject to greater pressure from the media regarding their body shape (31). Physical attractiveness also influences the way that an individual is treated. The so-called “halo effect” is when a person who possesses one positive feature is perceived as therefore having other positive features (38). Thus, individuals who would be considered unattractive or physically ugly are more likely to be perceived as having negative features. Even in children's stories, ugliness or a visible disability is often a marker of evil (39–41).

Fear of being unattractive, not fitting into the norms, or even being rejected due to one's looks may lead to serious mental disorders. Attitudes towards one's body are an important element of disorders such as anorexia and bulimia nervosa (42) as well as body dysmorphic disorder (43, 44). Shame and the sense of humiliation associated with the perception that one's environment can see one's flaws—skin problems, for example—and the fear of being rejected or isolated by “healthy” individuals may lead to mental health problems such as depression, addiction, or anxiety disorders (45). Individuals suffering from depression tend to focus more on their body mass and they may ruminate about their extra weight (46). There also exists a relationship between increased body mass and decreased mental health (47). Moreover the difference between actual (“real self”) and idealised (“ideal self”) body shape among young women increases emotional discomfort, anxiety, fear, and internal tension (48) Unattractive looks that do not “fit the norm” may thus be a major source of stigma (49).

A feature, mark, or social attribute may be the cause of deep stigmatisation. People suffering from such stigmas may be perceived as different, lesser, or even dehumanised (50). Unattractive looks and body imperfections (such as pimples, being too tall, too short, too fat, too skinny, etc.) are one of the most important sources of stigma, alongside character imperfections (laziness, mental illness, dishonesty) and identity (attached to identification with a particular race, ethnicity, religion, ideology, etc). Certain bodily imperfections (e.g., chronic skin diseases or prosthetic limbs) can be hidden, not immediately visible, and thus discreditable. In such situations, a person may decide whether and under what circumstances they will disclose their imperfection. A visible feature that cannot be disguised, such as height, obesity, or skin colour, is discredited because of its being clearly visible (51, 52). Someone who is thus stigmatised often experiences exclusion or rejection and their imperfection can become their defining attribute (53).

The difference between discredited and discreditable stigmas means that the assessment of attractiveness and the level of stigma differ depending on the context. Dermatological conditions and obesity are chronic health problems that impact one's sense of attractiveness, significantly lowering one's physical attractiveness and influencing quality of life (11, 54–58). However, a person with a visible skin condition (e.g., psoriasis or atopic dermatitis) is perceived differently than are obese (“fat”) individuals: the environment “blames” an individual for being obese (11). Psoriasis or atopic dermatitis are genetic conditions whose occurrence is outside the control of the person affected (59, 60). Psoriasis is a chronic disease that may undergo periods of remission, during which the condition improves and the associated problems may even disappear completely; however, it is impossible to completely cure it and prevent relapse (45). Obesity is a chronic disease characterised by the ratio between one's body mass and height squared being above 30 (61), and an individual's choices can play a role in the aetiology of the condition (it is commonly perceived that one can change their looks/body mass through exercise, diet, medication, or, in the most serious cases, by surgery). Thus, the stigma around obesity is two-fold: both the appearance (bodily imperfection) and character imperfection are stigmatised (49, 52).

Therefore, the goal of the current study was to investigate the role of subjective assessment of one's body in the relationship between objective indices of one's looks (body shape, body mass, presence of a skin condition) and the sense of stigma among young women.

To this end, the following hypotheses were formulated:

1. The level of perceived stigma will depend on the type of stigma: young women who are obese will experience the greatest sense of stigma due to being affected by a double stigma; the lowest levels of stigma will be experienced by the women in the control group.
2. Attitude towards one's body will be a moderator of the relationship between the objective parameters of the stigma-causing condition and the sense of stigma.

## Materials and Methods

### Participants

A total of 188 young adult women took part in the study (age: *M* = 25.58; *SD* = 2.90; min = 19, max = 30); we chose a young study group on purpose, as this is a developmental period in which body image is very important and assessments thereof are the most critical (19). We selected two groups of women who have conditions that affect how one looks: obesity and psoriasis. Despite the fact that these conditions often co-occur (62), we focused on groups of women with only obesity [as per WHO BMI ≥ 30, *n* = 54; (61)] or psoriasis (the inclusion criteria were at least 1 year having elapsed since diagnosis and visible skin lesions; *n* = 57). In order to assess whether the investigated phenomena occur only for the group with the visible stigmatising conditions, women with normal weight and no skin conditions were also included in the study (control group; *n* = 77). None of the study participants underwent bariatric surgery or laser skin therapy.

### Procedure

The recruitment procedure had two stages. During the first stage, females participating in a larger project, described elsewhere (63), who met the inclusion criteria for this study were recruited. During the second stage, females who met the inclusion criteria for this research project were asked to invite acquaintances to participate—i.e. a non-random method of sample selection [“snowball sampling technique”; (64)]. A total sample of 188 females was recruited for this study. Data were collected between 2018 and 2019.

Participants completed the following questionnaires: *the Perceived Stigmatisation Questionnaire, the Multidimensional Body-Self Relations Questionnaire*, and a short survey to collect medical and sociodemographic variables. These questionnaires were completed during a visit to a dietician, dermatologist, or psychologist, or at home (in this case, participants had 2 weeks to complete the questionnaires). Additionally, we collected objective body measurements: body mass, height, and sizes of individual body parts. This allowed us to calculate anthropometric indices for all participants, such as Body Mass Index (BMI), Index Of Central Obesity (ICO), and Waist-to-Hip Ratio (WHR). Moreover, the objective body parameters connected with body mass were controlled using a body composition analyzer. The data used for this study were part of a larger survey and the questionnaires that formed this study took around 25 min to complete.

The protocol of this study was approved by the Ethics Board for Research Projects at the Institute of Psychology, University of Gdansk, Poland (decision no. 12/2018).

#### Multidimensional Body-Self Relations Questionnaire

We used the Multidimensional Body-Self Relations Questionnaire (65), in its Polish adaptation (66), to measure the participants' body-image. This questionnaire is composed of 69 statements that assess the participants' attitudes towards their body's appearance. The scale has 10 subscales clustered into four areas: Appearance—*Appearance Evaluation* (AE), *Appearance Orientation* (AO), and *Body Areas Satisfaction* (BAS); Fitness—*Fitness Evaluation* (FE) and *Fitness Orientation* (FO); Health—*Health Evaluation* (HE) and *Health Orientation* (HO); *Illness Orientation* (IO); and Body Weight—*Overweight Preoccupation* (OP) and *Self-classified Weight* (SCW). Participants give their responses on a five-point Likert type scale ranging from 1 (*definitely disagree*) to 5 (*definitely agree*). The indicators are slightly different for some items: 1 (*never*), 2 (*rarely*), 3 (*sometimes*), 4 (*often*), and 5 (*very often*).

#### Perceived Stigmatisation Questionnaire

The Perceived Stigmatisation Questionnaire was used to assess sense of stigma in the young women (67). The questionnaire is composed of 21 items that form 3 subscales: *Absence of Friendly Behaviour, Confused/Staring Behaviour, Hostile Behaviour*, and *Total Score*. Participants assess on a five-point Likert-like scale how often people behave in certain ways around them, where 1 indicates *never*, 3 indicates *sometimes*, and 5 indicates *always*. In order to develop a Polish version of the PSQ, the questionnaire was translated into Polish independently by an interpreter and a psychologist with the author's consent. After selecting the best Polish version, it was back-translated into English by a native speaker. Then, the quality of translation was assessed by comparing the back-translation with the original questionnaire.

#### Sociodemographic Questionnaire

The sociodemographic questionnaire was composed of questions about age, height, marital status, place of residence (city, town, village), concomitant diseases, the diets one follows, as well as visits to specialist physicians and dieticians. With regards to dermatology, we asked about the severity of the condition and methods used to conceal or treat the visible skin lesions. In the part regarding body mass and shape, we used the body measurement data to calculate anthropometric indices.

#### Body Composition Analyzer

In order to measure the objective dimensions of the body and its components, we used the Segmental Body Composition Monitor–Tanita BC-601, produced by Tanita Corporation, Japan. BMI was calculated only as an inclusion criterion for the obesity group. The importance of body composition and visceral fat levels is increasingly emphasised (68, 69), which is why BMI was not used in further analyses. For adults, the analyzer allows the measurement of indices of obesity level adjusted for muscle mass content, fat percentage (%BF), recommended daily energetic intake, basal metabolic rate, metabolic age, bone mass, and visceral fat content (70).

#### Anthropometric Indices

Indices related to body shape were calculated based on a questionnaire prepared for the purposes of this study.

*Index of central obesity (ICO):* the ratio between waist circumference and height. This allows the assessment of the ratio between visceral fat and the total fat content. It is a more precise parameter for gauging one's health, because it takes visceral fat levels into account. It is also important for the parameters of one's body shape (71).

We also calculated the *Waist-to-hip ratio (WHR*), which is an index of body shape. It usually takes values between 0.6 and 1.0—the lower the index, the slimmer the waist in comparison to the hips (hourglass figure; (26, 72).

### Statistical Analysis

The analyses were performed in Python 3.8.5 programming language (Python Software Foundation, USA, 512 Lafayette Boulevard, Suite 2, Fredericksburg, Virginia 22401) programming language, using JupiterLab 2.2.6(Open Source Software) as the computation environment.

The following python libraries were used:

- pandas 1.1.3 (Open Source Software);
- scipy 1.5.2 (Open Source Software);
- numpy 1.19.2 (Open Source Software);
- pingouin 0.3.9 (Open Source Software).

Test data were contained in pandas.DataFrame. DataFrame is a two-dimensional, size-mutable, potentially heterogeneous tabular data type. Its structure contains labelled axes (rows and columns). Arithmetic operations align on both row and column labels. It can be considered a mathematical database. This study used DataFrame to index and align data. Each row contained all data collected from a single participant.

Wilk-Shapiro test was performed using scipy.stats.shapiro function. One way Anova test was performed using pingouin.anova function. Kurskal test was performed using scipy.stats.kruskal. Spearman's Rho correlations were calculated using scipy.stats.spearmanr function. Pearson's Rho correlations were calculated using scipy.stats.pearsonr function. Z-Score was calculated by executing pandas.DataFrame.apply(numpy.mstats.zscore). Moderation was calculated using python implementation of prof. Hayes, A. F. PROCESS macro. Wilk-Shapiro test was used to verify parametric assumptions on data used in the document.

As a result, non-parametric variables were found: Absence of Friendly Behaviour, Appearance Orientation (AO), Fitness Orientation (FO), Health Orientation (HO), Illness Orientation (IO).

For parametric variables, the validity of group selection was determined by using mean analysis, ANOVA, and Dunn's multiple comparison test. For non-parametric variables, the validity of group selection was determined by using mean analysis, Kruskal-Wallis *H*-test, and Tukey's range test.

Pearson Rho correlation ware calculated for parametric variables, between groups for Anthropometric Indices, PSQ, and MBSRQ while Spearman Rho correlations were calculated for non-parametric variables.

Bias-correct, no-nparametric, bootstrap PROCESS MACRO moderation was used to determine the relationship of Anthropometric Indices, and PSQ with MBSRQ as relation moderator.

## Results

### Objective Body Dimension Differences

First, we assessed whether groups had been selected appropriately. Their characteristics and anthropometric differences are presented in Table 1.

**Table 1:** Anthropometric differences between the groups.

**Anthropometric parameters**	**Control group (*****n*** **= 77)**		**Women with obesity (*****n*** **= 54)**		**Women with psoriasis (*****n*** **= 57)**		**ANOVA**			
	***M***	***SD***	***M***	***SD***	***M***	***SD***	***F***	***p***	**η^2^**	***DIFF***
Body mass	61.07	6.00	98.17	15.14	60.92	7.90	267.72	<0.001	**0.07**	O >C; O > P
BMI	21.74	1.64	35.01	4.14	22.04	2.21	442.46	<0.001	**0.08**	O >C; O > P
WHR	0.79	0.07	0.89	0.08	0.81	0.09	26.62	<0.001	**0.02**	O >C; O > P
ICO	0.45	0.04	0.63	0.07	0.47	0.05	221.03	<0.001	**0.07**	O >C; O > P
%BF	28.56	3.75	43.44	4.91	27.23	6.07	193.88	<0.001	**0.06**	O >C; O > P
Visceral fat	1.86	0.79	8.62	2.47	2.07	1.19	352.18	<0.001	**0.08**	O >C; O > P
Age	25.56	2.81	26.19	2.62	25.04	3.18	2.22	0.11	**0.02**	_
High	167.52	6.07	167.37	5.36	166.00	5.45	1.31	0.27	**0.01**	_
Disease severity	1.01	0.72	1.02	0.84	5.51	1.73	303.75	<0.001	**0.07**	O > C; P > O; P > C

*C, Control Group; O, Women with obesity; P, Women with Psoriasis; η^2^, magnitude of effect*.

The collected data indicate that the groups were selected appropriately. The groups differed in terms of the parameters that were key to the sampling process. Parameters regarding body mass and BMI were the highest in the group of women affected with obesity (Dunn *post hoc* analysis: *p* < 0.001), while those regarding the phase and the severity of disease were highest in the group affected by psoriasis (*p* < 0.001). The groups did not differ in terms of age and height.

### Participants' Attitudes Towards Their Own Body and Their Perceived Stigma

Attitudes towards one's own body differed between the groups (Table 2).

**Table 2:** Differences in the assessments of one's body (MBSRQ) between the groups.

**MBSRQ**		**Control group (*****n*** **= 77)**		**Overfat group (** ***n*** **=54)**		**Skin disease group (*****n*** **= 57)**		**ANOVA**			
		***M***	***SD***	***M***	***SD***	***M***	***SD***	***F***	***p***	***η^2^***	***DIFF***
Appearance	Appearance evaluation (AE)	21.62	3.10	19.26	4.62	21.60	3.47	19.10	<0.001	**0.01**	-
	Appearance orientation (AO)	39.61	4.85	38.67	6.04	39.51	5.24	2.76	0.25	**0.01**	O > C; O > P
	Body areas satisfaction (BAS)	31.21	5.61	26.69	5.80	30.47	5.94	20.40	<0.001	**0.10**	C > O; P > O
Fitness	Fitness evaluation (FE)	15.27	2.99	13.81	3.78	14.96	3.36	7.13	0.03	**0.03**	-
	Fitness orientation (FO)	35.04	3.48	35.69	5.40	35.04	4.22	0.43	0.65	**0.01**	-
Health	Health evaluation (HE)	18.95	2.31	18.57	3.21	19.65	2.13	6.57	0.04	**0.03**	C > O
	Health orientation (HO)	35.40	4.65	35.37	5.79	35.23	5.40	0.02	0.98	**0.01**	-
	Illness orientation (IO)	12.74	2.53	12.69	3.39	13.09	3.46	0.29	0.74	**0.01**	-
Body *Weight*	Overweight preoccupation (OP)	9.82	2.80	13.96	3.73	10.11	6.15	16.76	<0.001	**0.15**	O > C; O > P
	Self-classified weight (SCW)	6.30	1.36	9.31	0.86	6.05	1.67	101.09	<0.001	**0.05**	O > C; O > P

*C, Control Group; O, Women with obesity; P, Women with Psoriasis; η^2^, magnitude of effect*.

Women affected by obesity had the lowest satisfaction with their bodies (Tukey comparison with psoriasis: *p* > 0.002; and the control group: *p* = 0.001) and the lowest assessment of their looks (Dunn *post hoc* analysis: *p* > 0.001). It is not surprising that this was associated with the assessment of one's body mass (*post hoc* Tukey: *p* > 0.001) and with being preoccupied with one's weight as an aspect of the assessment of one's appearance (Dunn *post hoc* analysis: *p* < 0.001). Health, understood as an aspect of one's attitude one's own body, was assessed the highest by women with psoriasis (*post hoc* Tukey: *p* = 0.01).

Some statistically significant differences were also observed for perceived stigma (Table 3).

**Table 3:** Differences in perceived stigma between the groups.

**PSQ**	**Control group (*****n*** **= 77)**		**Women with obesity (*****n*** **= 54)**		**Women with psoriasis (*****n*** **= 57)**		**ANOVA**			
	***M***	***SD***	***M***	***SD***	***M***	***SD***	***F***	***p***	***η^2^***	***DIFF***
Absence of friendly behavior	2.24	0.39	2.19	0.45	2.08	0.47	5.25	0.07	**0.02**	P > C
Confused/staring behavior	1.70	0.37	1.97	0.62	1.64	0.43	7.75	<0.001	**0.08**	O > C; O > P
Hostile behavior	1.42	0.37	1.88	0.83	1.59	0.50	10.15	<0.001	**0.10**	O > C
Total score	1.84	0.29	2.03	0.49	1.79	0.35	6.27	0.002	**0.06**	O > C

*C, Control Group; O, Women with obesity; P, Women with Psoriasis; η^2^, magnitude of effect*.

Overall sense of stigma was greater in women affected by obesity than those affected by psoriasis (*p* = 0.012) or the control group (*p* = 0.044). Further analyses did not reveal any differences between women with psoriasis and women from the control group (*p* = 0.493).

### Objective Body Dimensions and Perceived Stigma

In the next step, we investigated the relationships of anthropometric indices and body dimension with perceived stigma. There was a surprising lack of relationship between perceived stigma and objective indices regarding body shape (WHR, ICO) as well as the severity of psoriasis (Disease Severity). However, there were correlations between sense of stigma and parameters associated with body mass and fat levels (Table 4). The greatest number of significant correlations were observed in the group of women affected by obesity. Interestingly, significant relationships revealed in the psoriasis group were negative, which means that the higher one's body mass, the lesser the perceived stigma (provided one does not reach the threshold of being overweight).

**Table 4:** Correlation between body dimension and perceived stigma.

		**Body dimension**		
**PSQ**	**Group**	**Body mass**	**%BF**	**Visceral fat**
**Absence of friendly behavior**	Control group (*n* = 77)	−0.22^*^	−0.31^**^	−0.16
	Women with obesity (*n* = 54)	0.06	0.10	0.08
	Women with psoriasis (*n* = 57)	−0.24	−0.24	−0.05
**Confused/** **staring behavior**	Control group (*n* = 77)	−0.07	−0.29^**^	−0.30^**^
	Women with obesity (*n* = 54)	0.40^**^	0.33^*^	0.39^**^
	Women with psoriasis (*n* = 57)	0.02	−0.08	0.16
**Hostile behavior**	Control group (*n* = 77)	−0.22	−0.24^*^	−0.12
	Women with obesity (*n* = 54)	0.48^***^	0.39^**^	0.36^**^
	Women with psoriasis (*n* = 57)	−0.31^*^	−0.18	0.00
**Total score**	Control Group (*n* = 77)	−0.22	−0.37^***^	−0.27^*^
	Women with obesity (*n* = 54)	0.41^**^	0.35^**^	0.36^**^
	Women with psoriasis (*n* = 57)	−0.21	−0.22	0.04

*** *p < 0.001,*

** *p < 0.01,*

* *p < 0.05*.

### Moderating Role of the Body Image for Perceived Stigma

**Figure 1:**
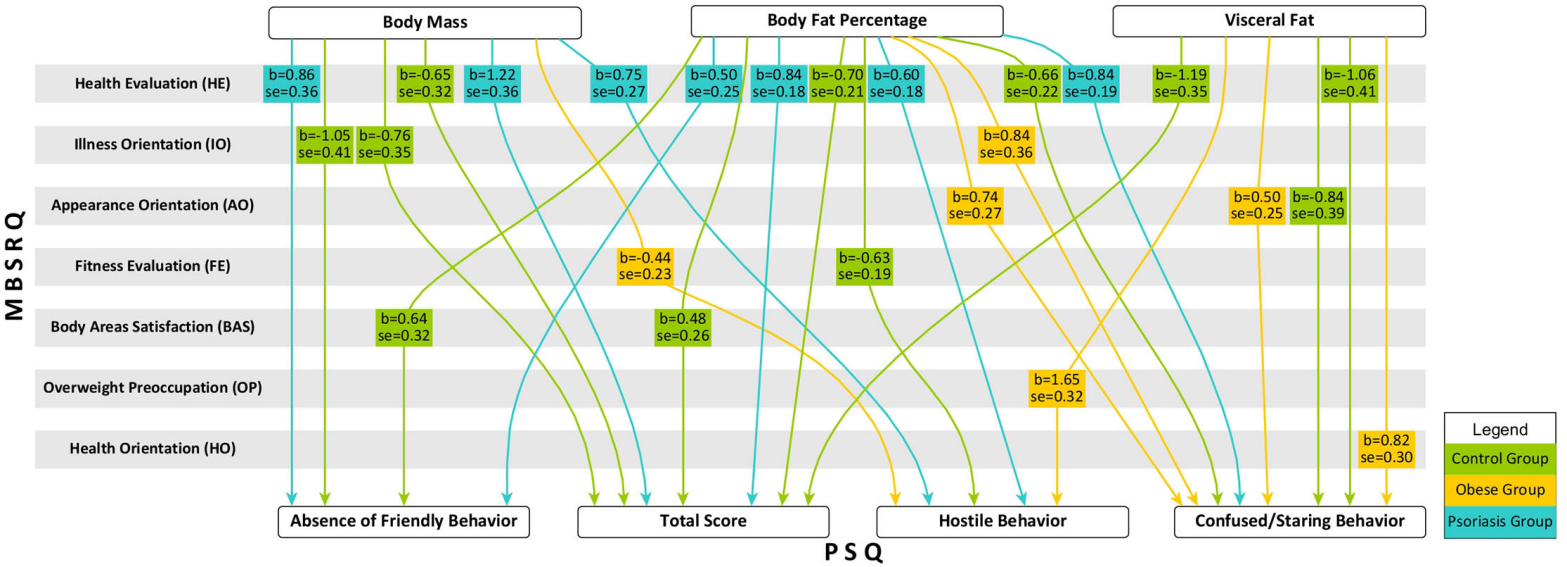
Moderation effect of body image on relation between objective body dimension and perceived stigma.

Moderation analysis was performed in order to investigate the role of subjective assessment of one's body in the relation between objective body measurements and perceived stigma (Figure 1).

No moderation effect was observed in the relation between objective indices regarding body shape (WHR and ICO) as well as the severity of psoriasis (Disease Severity) and perceived stigma. *Health Evaluation* was the most common moderator associated with the subjective attitude towards one's own body in the relationship between body dimensions (body mass, %BF, and visceral fat) and perceived stigma. It is worth noting that this was the only moderating factor in the group of women with psoriasis, while it did not moderate any of the relationships in the case of women with obesity. Interestingly, in the group of women affected with obesity, attitude towards one's own body significantly influenced the sense of being stared at and the experience of hostile behaviours in social relations when body mass or body fat levels were higher.

## Discussion

The main objective of this research was to verify the subjective role of the assessment of one's body in the relationship between objective indices of appearance and the perceived stigma among young women. The above-presented analyses largely supported the hypotheses.

### Sense of Stigma Among Young Women With Conditions That Decrease One's Physical Attractiveness

We hypothesised that the level of perceived stigma would depend on the type of stigma. The fact of being chronically ill itself, irrespective of the type of illness, results in stigma (73). However, our study found that not every illness leads to the same levels of perceived stigma. In line with the hypotheses, obese women were more likely to have a sense of stigma than women in other groups. This agrees with our previous results: a study on a smaller number of women (11) found that obese women were more likely to experience hostility as part of their stigma than women with skin conditions or those without an objective stigma. Lin et al. (74) asked 464 teenagers who were either overweight or non-obese about depressive symptoms, perceived stigma associated with body mass, and internalised stigma; they also measured actual and perceived weight status. They concluded that weight-related self-stigma is a problem even for people who are not overweight, because perceived weight stigma was associated with weight-related self-stigma regardless of body mass status. Strong associations between perceived weight stigma and weight-related self-stigma have been observed. In a study by Vartanian et al. (75), with 598 participants, pictures of women who were either obese or non-obese were shown to the participants, who assessed them with regards to emotions, attitudes, stereotypes, or desire for social distance. Images of obese individuals inspired more disgust and negative attitudes and stereotypes as well as greater desire for social distance. The researchers concluded that disgust plays an important role in prejudices and discrimination towards obese individuals and that it may partially explain prejudice against overweight people.

We also hypothesised that women affected with psoriasis would differ from the control group in terms of their perceived stigma; however, this hypothesis was not supported by the collected data. Moreover, the severity of the disease, its phase, and the size of affected skin surface also did not impact levels of perceived stigma. This is in line with the research by Rzeszutek et al. (76), which showed that some people affected by psoriasis do not differ in their attitudes from healthy individuals. Moreover, higher satisfaction with their own body and resources allows individuals with psoriasis levels of life satisfaction similar to those of the general population This is also in line with the research by Sakson-Obada and Wycisk (77), who found that accepting one's illness and positive body image and body experiences decreased the negative impact of psoriasis. Thus, positive body image may also facilitate more successful coping with physical symptoms of psoriasis and increase the well-being of patients (78), thereby decreasing perceived stigma.

Interestingly, women who were not overweight and did not suffer from any skin condition, even those with high body fat levels, did not perceive a lack of positive behaviours towards them or lack of compliments. This is in line with previous research (79) that found that women who were not overweight did not associate increased body mass or body fat with lack of friendly behaviours towards them. Contrasting results were observed by Lipowska et al. (33), where lack of perceived friendly behaviours was deemed an important component of stigma and compliments played an important role in building the body esteem of the young female participants, independently of their body mass.

### The Role of Attitudes Towards One's Body in the Relationship Between Objective Body Measurements and Perceived Stigma

We hypothesised that the attitude towards one's body would be a moderator of the relationship between the objective parameters of the stigmatising illness and perceived stigma. Attitudes towards one's body are developed from the earliest years of life, starting with the stage of getting to know one's own body. After that, these attitudes are shaped and internalised through comparisons with people around us, their opinions, and attitudes towards us. For girls and women, body image comprises a much bigger part of the “self” than is the case for men, and it strongly influences their overall self-esteem (35, 36). From the earliest years of life, women experience stigma and negative judgements associated with increased body mass. One study on a group of children aged 3 to 7 (80) in which participants were asked to describe their preferences for the appearance of playmates as well as to ascribe features attributed to normal and overweight figures revealed that girls are more strict in their judgements of overweight individuals than boys. As per Carof's report (81), despite the fact that European countries vary in their cultures' approaches to overfat and obesity, attitudes towards excess body mass are generally negative and are also often associated with moral judgements of the individual. This is why it is worth emphasising that our results indicate that body mass and its components (fat percentage and visceral fat) play an important role in the relationship with one's own body and perceived stigma. Independently of the objective stigma and body shape (proportions), preoccupation with one's body mass and control over it is a factor that influences one's body image and perceived stigma. Moreover, preoccupation with one's appearance was stronger in the group of obese women than in the remaining groups, and it played an important role in the relationship between objective body dimensions (body fat, visceral fat) and the sense of being stared at because of one's appearance. It can be thus supposed that weight is an important factor in determining to what extent a woman feels attractive, what attitudes she has towards her own body, and whether she perceives weight-related stigma. This is in line with the research of Tovée et al. (82), according to whom body weight explained as much as 53% of the variance in attractiveness among women, while in the case of men it was only 13%. Interestingly, according to the Faries and Bartholomew (83) study, fat percentage seems to be a strong indicator of attractiveness, and the influence of WHR and BMI on attractiveness is partially dependent on it. Despite the fact that multiple studies indicate that women are more preoccupied with their weight than are men (84) and women with higher body mass are more dissatisfied with their appearance (85–87), dissatisfaction with one's appearance and body mass appears to be a universal phenomenon for women (88). Similar results were observed in a study in which all female participants, independently of their body mass and body fat levels, expressed dissatisfaction with their body mass and a desire to reduce it (79). Interestingly, in the study Blodorn et al. (89), women with higher weight, when they were to describe during the study why they would make a good date, and their potential partner would see or hear their recording, felt greater expectations of social rejection when weight was seen (vs. unseen). Experimental studies (90) additionally revealed that showing women who are initially dissatisfied with their bodies images of a very slim “perfect” body significantly increased their levels of dissatisfaction, and led them to judge themselves more harshly. This effect was not observed in women who were initially satisfied with their bodies. Body weight is therefore one of the strongest predictors of satisfaction with one's appearance in women - the lowest level of satisfaction is shown by people with the highest body mass (91).

Moreover, our analyses did not support the existence of a relationship between parameters related to body shape and perceived stigma when the subjective assessment of one's body was tested as a moderator of this relationship. Similar results were observed for a group of Polish and Vietnamese young people (33), for whom objective body measurements did not influence sense of stigma among women. No relationship between anthropometric indices and assessment of one's own body was observed and waist to hip ratio was associated with one's satisfaction with body mass and physical fitness. Moreover, in Polish women, BMI had little influence over assessment of their bodies. This again supports the claim that body mass and its components, and not body shape, influences our attitudes towards our own bodies, and whether we perceive stigma related to our appearance.

A surprising result of the presented study was the positive assessment of one's own health by women affected with psoriasis. This positive assessment seems to lower the levels of perceived stigma, even when body mass or body fat levels are higher. This is in line with the results of a study by Alexandrova-Karamanova (92), who found that young people who assessed their health positively also exhibited more positive feelings and attitudes towards their bodies and appearance. This is even more surprising because psoriasis significantly impacts one's sense of physical attractiveness and acceptance of one's own body (77). A completely different approach towards one's own body and stigma was revealed in the case of obese women. It is likely that women do not treat obesity as an illness *per se*. Obesity is associated with a number of consequences for one's physical health (93–95); however, these medical conditions are often not directly associated with obesity (in the sense that people not affected by obesity also suffer from these conditions). Thus, it is possible that an individual does not perceive such diseases as associated with obesity, but rather as conditions independent of their appearance, which is why they assess their health differently and pay less attention to the evaluation thereof in the context of their appearance or stigma. Skin conditions require treatment on many levels, such as taking good care of one's skin, alleviating itchiness, regular visits to a specialist, and concealing the affected areas (96, 97). In the case of psoriasis, believing that one has internal control over one's health is associated with the acceptance of the condition, which facilitates adherence to guidelines given by specialists, which, in turn, allows for relatively successful treatment of symptoms (98). Thus, a positive assessment of one's health may allow one to better cope with the physical symptoms of psoriasis and helps reduce stress (which exacerbates the condition) (99).

## Conclusion

Summing up, the main conclusion of our study is the fact that subjective assessment of one's body and attitudes towards one's body influence perceived stigma, independently of objective body shape or the type of condition causing the stigma. At the same time, body mass is a strong predictor of perceived stigma for all women.

Levels of perceived stigma differ between women with psoriasis and women affected by obesity. Obese women feel stigmatised more strongly than the rest of the group. Patients suffering from psoriasis assessed their health as most inportnant element of body evaluation, which may result in a lower sense of stigmatisation in this group. Therefore, perceived stigma should be taken into account when working with obese individuals and actions could be taken to help improve their body image. Furthermore, social programmes should take the necessary steps to reduce stigma related to body mass, through prophylaxis in the field of health, sugar and lipid economy, physical activity as well as psychological and medical support. When working with stigmatised women, it is also worth focusing on their resources and skills, which will increase their self-esteem and self-efficacy. At the same time, as long as the media portrays very slim female bodies with smooth skin as desirable, comparing one's own body to that model will lower body satisfaction and foster eating disorders.

## Limitations

Our research involved young women who are particularly vulnerable to social disapproval and criticism for having an unattractive appearance and appearance that does not meet age norms. Moreover, as we mentioned, body image is a much more important element of “Self” for girls and women than for men. At the same time, it would be worthwhile to also study young men affected by obesity and psoriasis in order to assess the role that gender plays in these phenomena. This will broaden our understanding of the role of body image in the sense of stigma in those affected by appearance-related diseases. Another limitation is related to the selection of participants based on just two medical conditions: it is possible that extending the study to other visible conditions that may cause stigma could reveal more general tendencies regarding the relationship between one's body, attitudes towards one's body, and perceived stigma.

The sample size is another limitation of our study. Despite the fact that both excess body mass and skin conditions affect a large number of women, not all the women who were invited to take part in the study agreed to the collection of anthropometric data (e.g., stepping on the scale). Therefore, the results of our study cannot be generalised to all women with obesity and psoriasis. The sample size also limits the number and depth of analyses that could be conducted. While we can correlate anthropometric variables with sense of stigma, a longitudinal study would allow us to better determine causality. It would be worthwhile to extend the study to see whether similar results can be observed in a bigger sample. The way that participants were recruited (deliberate sample selection) may also be a limitation. Moreover, place of residence, marital status, and parental status were not taken into account in the study, thereby limiting the possible analyses. It may be that pregnancy or place of residence influence the perceived stigma or the attention paid to particular body imperfections.

## Data Availability Statement

The raw data supporting the conclusions of this article will be made available by the authors, without undue reservation.

## Ethics Statement

The studies involving human participants were reviewed and approved by the Ethics Board for Research Projects at the Institute of Psychology, University of Gdansk, Poland (decision no. 12/2018). Written informed consent for participation was not required for this study in accordance with the national legislation and the institutional requirements.

## Author Contributions

NM and ML: conceptualisation, methodology, writing—original draft preparation, and writing—review and editing. JK: software and validation. JK and ML: formal analysis. NM: investigation, data curation, and project administration. ML: supervision and funding acquisition. All authors contributed to the article and approved the submitted version.

## Funding

The work of ML was supported by grant 2015/17/B/HS6/04144 from the National Science Centre, Poland; the University of Gdansk covered the costs of open access publishing.

## Conflict of Interest

The authors declare that the research was conducted in the absence of any commercial or financial relationships that could be construed as a potential conflict of interest.

## Publisher's Note

All claims expressed in this article are solely those of the authors and do not necessarily represent those of their affiliated organizations, or those of the publisher, the editors and the reviewers. Any product that may be evaluated in this article, or claim that may be made by its manufacturer, is not guaranteed or endorsed by the publisher.
